# An aptamer and Au/Si CCA based SERS sensor for ultra-sensitive detection of Vimentin during EMT in gastric cancer

**DOI:** 10.3389/fbioe.2023.1310258

**Published:** 2023-12-07

**Authors:** Lingling Cheng, Jianlin Xu, Hua Yuan, Qihao Zhao, Wei Yue, Shuang Ma, Weimin Lu

**Affiliations:** ^1^ Department of Oncology, Yancheng TCM Hospital Affiliated to Nanjing University of Chinese Medicine, Yancheng, Jiangsu, China; ^2^ Pharmacy Department, Yancheng TCM Hospital Affiliated to Nanjing University of Chinese Medicine, Yancheng, Jiangsu, China; ^3^ Department of Laboratory Medicine, Yancheng TCM Hospital Affiliated to Nanjing University of Chinese Medicine, Yancheng, Jiangsu, China; ^4^ General Internal Medicine, Affiliated Hospital of Nanjing University of Chinese Medicine, Jiangsu Province Hospital of Chinese Medicine, Nanjing, Jiangsu, China

**Keywords:** Au/Si CCA, epithelial-mesenchymal transition, gastric cancer, surface-enhanced Raman scattering, Vimentin

## Abstract

**Introduction:** In this study, a surface-enhanced Raman scattering (SERS) sensor based on a functionalized Au/Si cap-cone array (Au/Si CCA) was constructed using the identity-release strategy to detect Vimentin changes during epithelial-mesenchymal transition (EMT) in gastric cancer (GC).

**Methods:** The periodic structure of Au/Si CCA, which can form “hot spots” with high density and regular arrangement, is a substrate with excellent performance. Au/Si CCA was functionalized with aptamers as the capture substrate, and Au nanocubes (AuNCs) were modified with 5-carboxyfluorescein (5-FAM) labelled complementary strand as SERS probe. The capture substrate and SERS probe were assembled by hybridization, and the SERS signal intensity of 5-FAM was greatly enhanced. The binding of Vimentin to the aptamer resulted in a broken connection between the SERS sensor Au/Si CCA array and AuNCs, which resulted in a decrease in the signal intensity of 5-FAM. The identity-release strategy requires only a simple step of reaction to achieve rapid detection of target proteins, which has clinical practicability.

**Results:** Using this protocol, the concentration of Vimentin in GES-1 cells could be successfully detected, and the detection limit was as low as 4.92 pg/mL. Biological experiments of Vincristine, Oncovin (VCR)-treated GES-1 cells effectively mimicked the EMT process, and Vimentin changes during EMT could be accurately detected by this method.

**Discussion:** This study provides a selective, ultra-sensitive and accurate assay for Vimentin detection, which may provide a means for the future detection of EMT process in GC.

## 1 Introduction

Gastric cancer (GC) is one of the most common and high-risk malignant tumors in the digestive system (Li et al., 2020). It originates from mucosal epithelial cells on the inner surface of the gastric wall and can occur in various parts of the stomach (Liang et al., 2023; Liu et al., 2023). The onset of GC is relatively hidden and the early symptoms are not typical, leading to many patients usually being diagnosed in the late stage (Monster et al., 2022). GC has the characteristics of easy metastasis and recurrence (Wei et al., 2017). GC patients are often accompanied by lymph node metastasis, peritoneal metastasis and liver metastasis, so their prognosis is extremely poor (Mita et al., 2023). Infiltration and metastasis of tumors are one of the most common causes of death. Metastasis is a complex multi-step process and epithelial-mesenchymal transition (EMT) is a key step in GC metastasis (Mahmoudian et al., 2023). The transformation of adherent epithelial cells into mesenchymal cells during EMT enables the cells to acquire powerful pro-tumor properties, including motility, aggressiveness, dryness, the ability to form metastasis and drug resistance (Zhang Z. et al., 2023b). The abnormal activation of EMT plays an important role in the invasion, metastasis and recurrence of GC (Pretzsch et al., 2022; An and Liu, 2023; Eddin et al., 2023). At present, GC has become a major burden on society, early detection of the key factor in the EMT process and subsequent treatment will improve patient prognosis.

Vimentin is a cytoskeletal intermediate filament protein, which is mainly expressed in mesenchymal origin tissues and the cytoplasm of mesenchymal tumor cells, and plays an important role in epithelial cell development, incision healing and tumor invasion and metastasis (Paulin et al., 2022). Expression of waveform proteins in epithelial cells is critical for continuous EMT through interactions with actin and other intermediate filaments. Waveform protein expression in epithelial cells has been reported to be associated with the malignant phenotype of cancer cells. Also patients with waveform protein-positive gastric cancer have significantly worse prognosis than patients with waveform protein-negative gastric cancer, and waveform protein expression may be a useful biomarker for determining the biological aggressiveness of gastric cancer (Brzozowa et al., 2015). In addition waveform protein expression in epithelial cells is crucial for EMT, which is associated with the acquisition of invasive properties of cancer cells (Yin et al., 2018). Several studies related to GC have shown that Vimentin can induce EMT and promote the invasion and metastasis of GC cells (Keyghobadi et al., 2022; Wang et al., 2023). It can be used as an important marker in EMT process (Yin et al., 2018). In the past, Vimentin was detected by immunological, electrochemical and fluorescent methods (Fhied et al., 2014; Wei et al., 2020; Peng et al., 2023). These methods have drawbacks such as susceptibility to interference, time-consuming, high cost, and complex to operate. Therefore, it is crucial to find a rapid and simple detection method with high sensitivity and specificity.

To address the above issues, an aptamer composed of nucleic acids was introduced as a molecular recognition probe. Aptamers are artificial short single-stranded oligonucleotides of DNA or RNA selected by Systematic Evolution of Ligands by Exponential Enrichment (SELEX) (Tuerk and Gold, 1990). Aptamer has excellent chemical stability and is not easily limited by the environmental conditions of the biosensing method (Liu et al., 2022; Nourizad et al., 2023). Aptamers are linked to complementary DNA or RNA strands by hybridization, a signaling strategy guided by preprogrammed Watson-Crick base-pairing, to form double-stranded structures called duplexed aptamer (DA), which regulate aptamer function (Nutiu and Li, 2003). In DA, the aptamer acts as a ligand-binding agent, whereas the complementary strand, which initially hybridizes to a defined portion of the aptamer, acts as a competing binding agent and generates signals during ligand-dependent dehybridization (Munzar et al., 2018; 2019). Conformational selection assumes that the complementary chain is first dehybridized from the DA to produce a free aptamer, which is able to fold into a three-dimensional structure and bind with high affinity and specificity to a particular target molecule (Song et al., 2008). The development of sensors based on the advantages of aptamers such as simplicity, speed, low cost, high sensitivity and high specificity has attracted much attention (Arishi et al., 2023; Gao et al., 2023; Park et al., 2023).

Surface-enhanced Raman scattering (SERS) can identify molecular “fingerprint” information, parameters such as characteristic peak position and intensity of the spectral signal can reflect the composition and structure of functional groups and chemical bonds in the molecule, thus achieving the specific detection of the molecule (Gu et al., 2023). Precious metal materials (Au, Ag, Cu) are the strongest metal materials in SERS effect, and their morphology, size and aggregation state have great influence on SERS effect (Yang et al., 2018; Sai et al., 2023).

Au/Si CCA, when used as a SERS substrate, ensures the uniform distribution of hot spots and adsorbed target molecules on the chip, enabling excellent signal reproducibility and accurate quantitative SERS detection. The edges and corners of AuNCs generate significantly enhanced localized electromagnetic fields on individual nanoparticles or between coupled nanoparticles, which can lead to significant SERS enhancement (Park et al., 2018). Therefore, SERS technology has the advantages of high sensitivity, good specificity, simple and fast operation (López-Lorente, 2021). Pan et al. (2022) developed a sensitive and direct SERS aptamer sensor to detect exosomes. The as-fabricated SERS aptasensor was capable of detecting exosomes in a wide range from 55 to 5.5 × 10^5^ particles μL^−1^ with a detection limit of 17 particles μL^−1^. Zhang et al. designed a ratiometric SERS biosensor for highly sensitive detection of exosomes that accurately identifies breast cancer cell-derived exosomes in clinical serum samples with ultra-low detection limits as low as 1.5 × 10^2^ particles/mL (Zhang Q. et al., 2023a). Zhao developed a novel SERS-based aptasensor to detect prostate-specific antigen biomarkers with an The limit of detection (LOD) is 6 pg/mL (Zhao et al., 2022). Thus, by combining SERS with aptamers, the sensitivity and accuracy of the assay were significantly improved.

**FIGURE 1:**
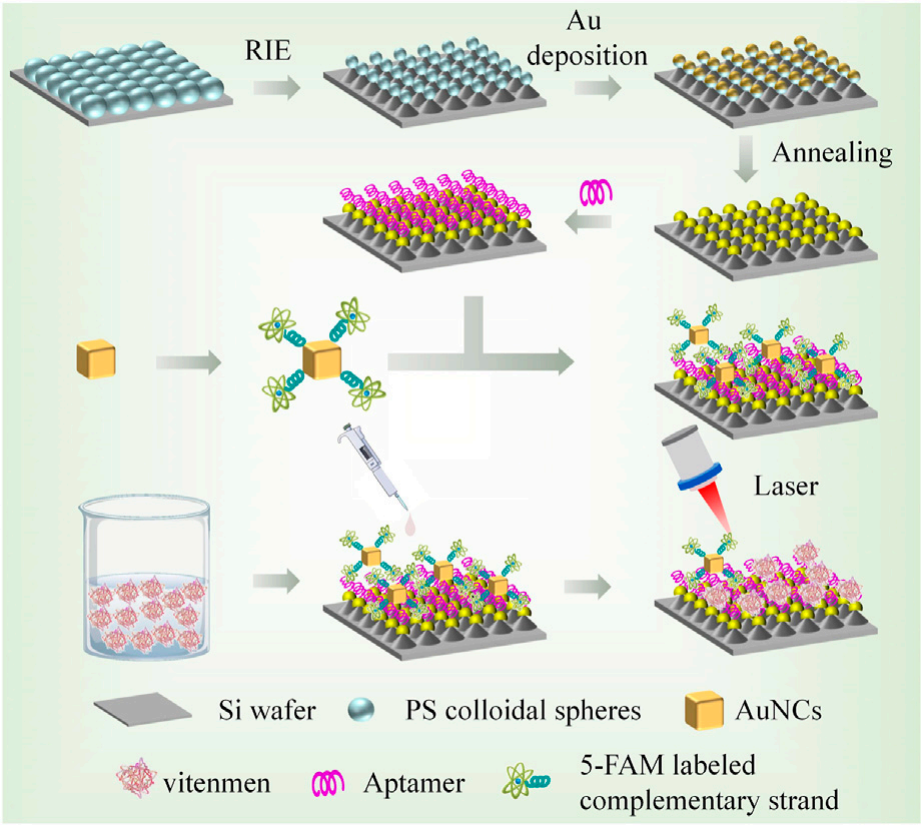
Preparation of capture substrate and SERS probe and schematic diagram of SERS sensor for Vimentin detection.

Herein, a novel SERS sensor based on identity-release strategy, Au/Si cap-cone array (Au/Si CCA) and Au nanocubes (AuNCs) were constructed. In this study, aptamer functionalized Au/Si CCA was used as the capture substrate, and AuNCs was combined with 5-carboxyfluorescein (5-FAM) labelled complementary strand as the SERS probe. When the target Vimentin was present on the capture substrate, the aptamer specifically recognizes the target protein and stably bound to it. At this time, the SERS probe was moved away from the substrate due to the competition of the target, resulting in the reduction of hot spots and the weakening of the Raman signal. Use this SERS sensor to detect and collect Raman signal from 5-FAM. Analyzing its data can qualitatively and quantitatively reflect the concentration of the target object. The schematic diagram is shown in Figure 1. The SERS sensor has the following advantages. First, Au/Si CCA can form a high density and uniform “hot spot” due to its periodic arrangement structure, so that it has excellent SERS performance. Second, the identity-release strategy was selected to significantly improve the specificity. Third, in addition to excellent sensitivity and specificity, the sensor also has the advantages of simple operation, short time consumption and strong anti-interference. These advantages showed the excellent performance of the SERS sensor in the detection of EMT in GC.

## 2 Experimental section

### 2.1 Materials

Hexadecyl trimethyl ammonium bromide (CTAB), chloroauric acid tetrahydrate (HAuCl_4_), sodium borohydride (NaBH_4_), ascorbic acid (AA), Mercaptobenzoic acid (4-MBA), Absolute ethanol, 6-Mercapto-1-hexanol (6-MCH), Vincristine, Oncovin (VCR), and polystyrene sphere (PS) suspension were all purchased from Sinopharm Chemical Reagent Suzhou Co. Sulfur hexa-fluoride (SF_6_) etching gas from Wuhan NewRead Specialty Gases Co. Glycerin, Phosphate buffer, and the whole protein extraction kit were purchased from China Solaibo Co. Australian Fetal Bovine Serum was purchased from Thermo Fisher Scientific (China) Co. Antibodies to Vimentin, E-Cadherin, N-Cadherin, C-Reactive Protein, Ki67, and ACTIN were purchased from Beijing Boosun Biotechnology Co. BSA, paraformaldehyde, PBS, trypsin, FPS, and DMEM were purchased from Gibco; Antibody dilution, BCA protein concentration kit, penicillin-streptomycin solution were purchased from China Biyuntian Biotechnology Co. Ultrapure water is obtained through Milli-Q purifiers (resistivity>18 MΩ cm); GES-1 cells were obtained from the School of Clinical Medicine of Yangzhou University; Table 1 shows the DNA sequences utilised in this work, which were bought from Shanghai Sangong Biological Engineering Co.

**TABLE 1:** The sequence of Vimentin aptamer and complementary strand.

Name	Sequence (5′–3′)
Aptamer	SH-TAGACCCAGCTGGTCCGGAAAATAAGATG
	TCACGGATCCTC
Complementary strand	SH-GAGGATCCGTGACATCTT-5-FAM

### 2.2 Instrumentation

S-4800 Ⅱ Field emission scanning electron microscope (SEM, Hitachi, Japan); Tecnai G2 F30 Field emission transmission electron microscope (TEM, FEI, United States); Cary UV-5000 Ultraviolet absorption spectrometer (Agilent, United States); InVia Reflex microscopic Raman spectrometer (Renishaw, United Kingdom); DXR3xi Raman imaging microscope (Thermo Fisher, United States).

### 2.3 Fabrication of functionalized Au/Si CCA

Au/Si CCA is fabricated by PS colloidal sphere template-assisted reactive ion etching (RIE) process and combined with magnetron sputtering deposition technique. Firstly, a layer of tightly stacked PS colloidal spheres was laid flat on a silicon wafer with an area of 5 × 5 cm by the Langmuir-Blodgett (L-B) film method, which was placed in an oven at 70°C for 20 min, so that the colloidal sphere template could be in close contact with the silicon wafer. Subsequent etching of PS colloidal sphere template-covered wafers in RIE machine using SF_6_ plasma (Power 120 W, gas flow rate 50 sccm). After 150 s of etching, highly ordered arrays of silicon cones were formed, with PS colloidal spheres at the top that shrank residually after etching. Notably, a cap-like Au layer was deposited on the PS colloidal spheres at the top of the silicon cone arrays using a magnetron sputtering apparatus at a sputtering rate of 1 nm/s and finally rinsed with ethanol and annealed for 2 h to remove the residual PS colloidal spheres.

### 2.4 Synthesis of AuNCs

First, mixing CTAB (7.5 mL, 0.2 mol/L), HAuCl_4_ (0.5 mL, 0.01 mol/L), and 7.5 mL of ultrapure water in a clean beaker. The solution was stirred vigorously (900 rpm) at 30°C before adding ice NaBH_4_ (1.2 mL, 0.01 mol/L), which was changed from colorless to brown after stirring was continued for 5 min. The solution was then allowed to stand at 30°C for 2 h. Thus, the Au seed solution was made. The prepared Au seed solution was diluted 100-fold and set aside.

CTAB (7.2 mL, 0.1 mol/L), HAuCl_4_ (0.18 mL, 0.01 mol/L), AA (2.7 mL, 0.1 mol/L), and 35 mL of ultrapure water were added sequentially in a beaker and stirred well. After 0.6 mL of the Au seed solution was added, the solution of Au octahedral seeds was obtained by vigorously stirring for 10 min and then left for 12 h. Subsequently, HAuCl_4_ (0.75 mL, 0.025 mol/L) was added to the beaker, mixed well, and left for 2 h to obtain AuNCs. After centrifugation twice to remove the supernatant, it was dispersed in CTAB and stored.

### 2.5 Assembly of Vimentin SERS sensors

The Vimentin aptamer and complementary strand were assembled on Au/Si cap-cone arrays and AuNCs, respectively, referring to the freeze-thaw cycle assembly method of Lou et al. (Lou et al., 2011). In concrete terms, after the Vimentin aptamer and the complementary strand of the aptamer were added to ultrapure water to prepare the solutions (1 mmol/L), 100 mL of the aptamer solution with Au/Si cap-cone arrays were soaked in centrifuge tubes, and 150 mL of the aptamer complementary strand solution was mixed with AuNCs into centrifuge tubes. Then, they were placed in the refrigerator (−20°C) to freeze for 2 h simultaneously before being taken out to thaw. Next, 150 mL of 6-MCH was added to each centrifuge tube and reacted for 2 h. Afterward, the Vimentin aptamer product and the aptamer complementary strand product were water-bathed for 5 min at 95°C, and finally, the two were mixed and reacted for 4 h.

### 2.6 SERS testing

GES-1 cells were cultured in DMEM medium containing 10% FBS and dual antibodies (penicillin 100 U/mL, streptomycin 100 μg/mL) in an incubator at 37°C and 5% CO_2_. Cellular proteins were extracted according to the instructions of the whole protein extraction kit and stored in a −80°C refrigerator. Vimentin protein was dissolved in aqueous glycerol solution and configured into a solution of 100 mg/mL, which was added to the protein extract, and the Vimentin protein spiked concentration was obtained by gradient dilution as 100 pg/mL–100 mg/mL. The droplet to be measured was added to the prepared SERS sensor for reaction for 10 min, then dried, and then SERS detected. The Raman spectrometer was used to select a 785 nm excitation laser with a laser power of 5 mW, and the acquisition time of each spectrum was 10 s.

## 3 Results and discussions

### 3.1 Characterization of Au/Si CCA

**FIGURE 2:**
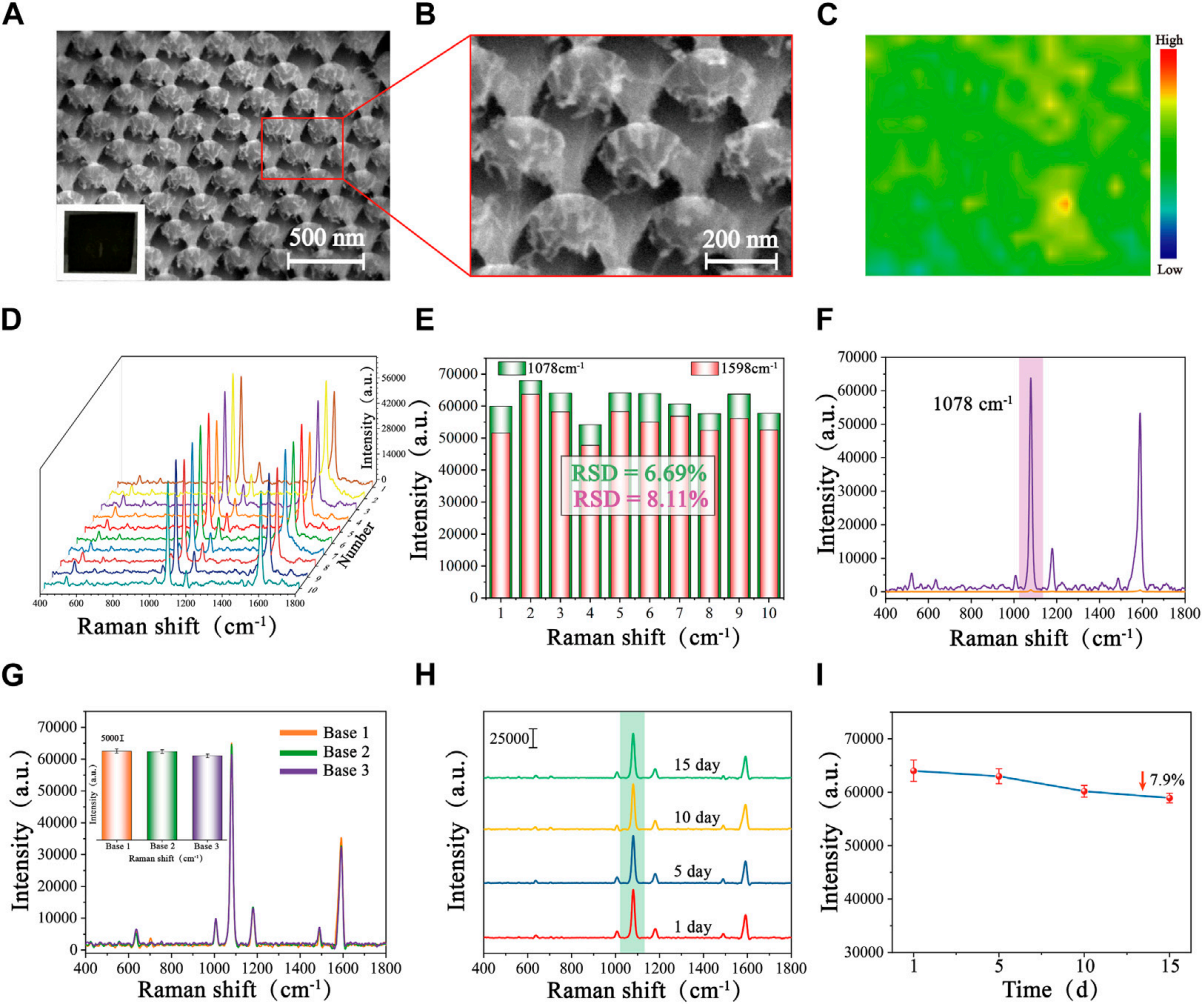
SEM images of Au/Si CCA with different magnifications: **(A)** Low magnification, and **(B)** High magnification. **(C)** SERS mapping of Au/Si CCA modified with 4-MBA (1 × 10^−8^ M). **(D)** SERS spectra of 10 randomly selected points on the surface of the 4-MBA-labelled Au/Si CCA and **(E)** the corresponding histogram of the intensity at 1,078 cm^−1^ and 1,598 cm^−1^. **(F)** SERS spectra of Au/Si CCA were modified with 4-MBA (1 × 10^−8^ M) and pure 4-MBA (1 × 10^−1^ M). **(G)** SERS spectra of 4-MBA-labelled Au/Si CCA prepared in different batches and the corresponding histogram. **(H)** SERS spectra of 4-MBA-labelled Au/Si CCA were stored at room temperature for 1 day, 5 days, 10 days, 15 days, and **(I)** the corresponding line graphs of the intensity at 1,078 cm^−1^.

From the SEM images and localized magnified images of Au/Si CCA (Figures 2A, B), it could be seen that the Au/Si CCA from a perspective was arranged in an ordered periodicity with a period of 200 nm, corresponding to the diameter of the PS colloidal spheres. The single Au/Si cap-cone could visibly observe the Au cap structure, whose superior specific surface area could provide abundant binding sites for the immobilization of biomolecules, facilitating the follow-up functionalization. To assess the signal homogeneity of the Au/Si CCA, 4-mercaptobenzoic acid (4-MBA) was added dropwise as a Raman signal molecule on the surface of the arrays, which was then allowed to dry naturally before being subjected to SERS mapping, with the scanning range set to 50 × 40 mm. The color change was utilized to illustrate the SERS signal strength at 1,078 cm^−1^ (blue is the lowest, and red is the highest). Figure 2C reveals that the color distribution of the SERS-mapped image of the array is regular, indicating its excellent uniformity. Furthermore, 10 sites on the Au/Si CCA surface were randomly chosen for spectral detection to obtain the average spectra of the characteristic peak intensities at 1,078 cm^−1^ and 1,598 cm^−1^ (Figures 2D, E), and the obtained SERS spectra were similar in shape, with relative standard deviations (RSD) of the signal intensities at 1,078 cm^−1^ and 1,598 cm^−1^ being only 6.69% and 8.11%, demonstrating the benefits of the array’s high uniformity. The enhancement factor (EF) of Au/Si CCA was calculated to be 9.2 × 10^8^ based on the SERS spectra recorded in Figure 2F, employing the formula: EF = (I_SERS_/C_SERS_)/(I_R_/C_R_), where I_SERS_ and I_R_ were the intensities obtained from 4-MBA-modified Au/Si CCA and silicon wafers, respectively, at 1 × 10^−8^ M and 1 × 10^−1^ M concentrations. Next, to assess the reproducibility of the array, three batches of Au/Si CCA were prepared at different times and subjected to SERS measurements. The average spectrogram and histogram of the intensity of the characteristic peaks at 1,078 cm^−1^ are shown in Figure 2G, with only minor differences in the shapes and intensities of the spectra, indicating that the Au/Si CCA has decent reproducibility. The stability of the arrays is crucial for subsequent applications, so the same batch of Au/Si CCA was stored at room temperature for 1 day, 5 days, 10 days, and 15 days to evaluate the stability of the arrays (Figure 2H). As shown in Figure 2I, the SERS signal intensity collected with Au/Si CCA stored for different days was not significantly reduced, which only decreased by 7.9% after 15 days of storage compared to the first day, indicating that the Au/Si CCA prepared in this study is very stable.

### 3.2 Characterization of AuNCs

**FIGURE 3:**
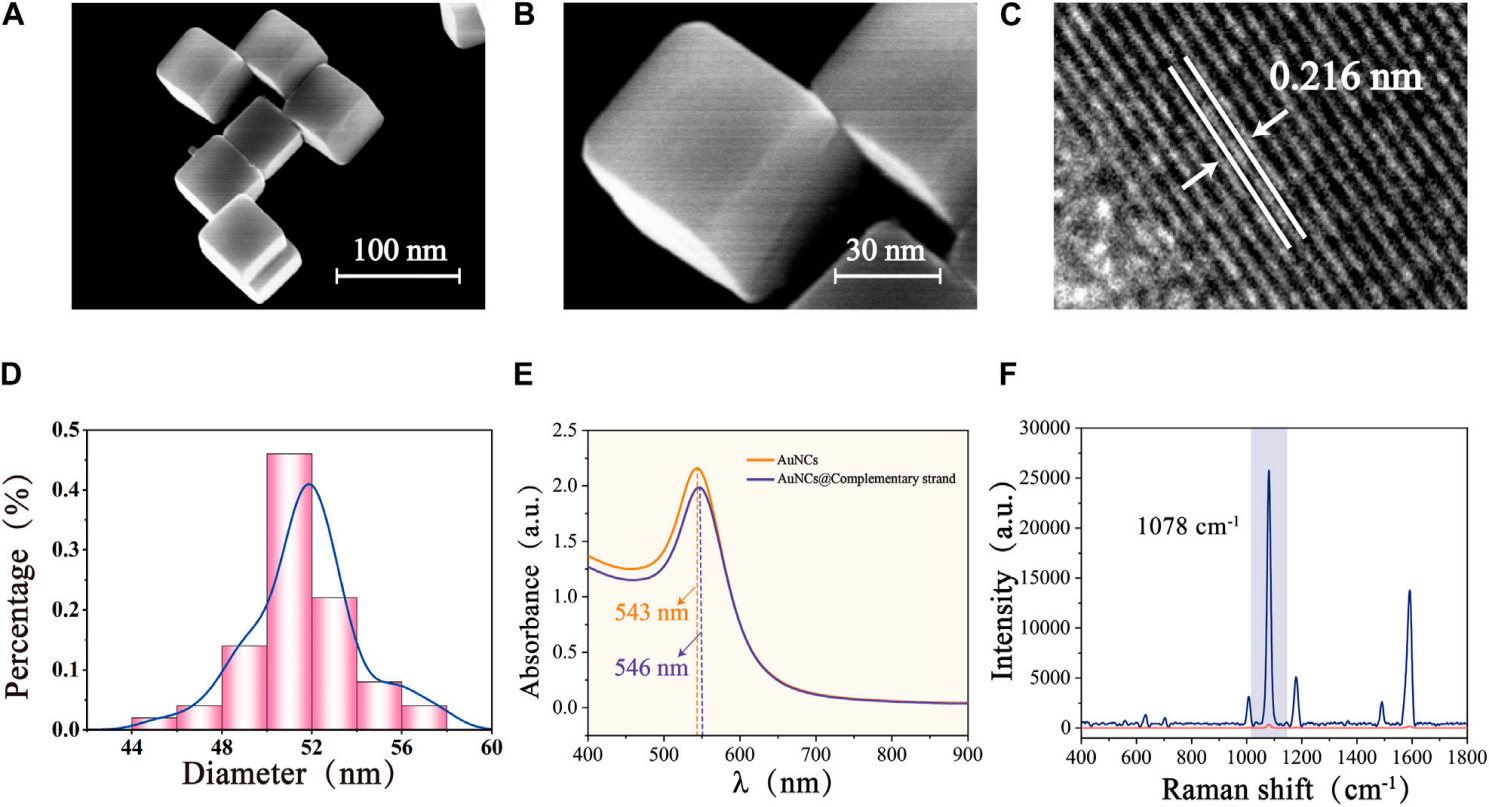
SEM images of AuNCs with different magnifications: **(A)** Low magnification, and **(B)** High magnification. **(C)** HRTEM image of AuNCs. **(D)** Size distribution of AuNCs. **(E)** UV-Vis-NIR spectra of AuNCs and AuNCs@ complementary strand. **(F)** SERS spectra of pure 4-MBA (1 × 10^−2^ M) and 4-MBA-labelled Au- AuNCs (1 × 10^−7^ M).

The low magnification SEM image (Figure 3A) showed that the AuNCs synthesized by the seed-mediated growth method were uniform in shape and size, well dispersed, and characterized by a typical cubic structure. The sharp vertices and edges could be clearly observed under high magnification (Figure 3B), which served as a carrier for a large number of “hot spots,” ensuring excellent SERS enhancement. The HRTEM image of the sample (Figure 3C) obtained following centrifugation to remove a significant quantity of the protective CTAB and drop it onto a copper mesh, which demonstrated a lattice spacing of 0.216 nm for AuNCs, coinciding with the growth interface of gold crystals. As a precious metal nanomaterial, gold nanoparticles possessed excellent stability and superior optical properties. Figure 3D showed a histogram of the particle size of AuNCs (50 AuNCs were randomly selected by Nano Measurement software, and their particle sizes were counted individually), calculating that the average particle size of AuNCs and its deviation was 51.73 ± 3.775 nm, with good size uniformity. Meanwhile, UV-Vis-NIR spectra showed a visible absorption band at 543 nm. Due to the 5-FAM labelled complementary strand modification, the maximum wavelength peak of AuNCs has a slight redshift at 3 nm, along with a decrease in the SERS signal intensity (Figure 3E). This may be attributed to the reduction of interstitial hotspots between neighboring AuNCs due to the interaction between the 5-FAM labelled complementary strand and AuNCs. This result surfaces that the 5-FAM labelled complementary strand has been successfully modified to the surface of AuNCs. The computed EF of AuNCs modified with 4-MBA (1 × 10^−7^ M) was 7.3 × 10^6^, demonstrating outstanding SERS performance and aiding in the Vimentin SERS detection (Figure 3F).

### 3.3 Experimental feasibility analysis

**FIGURE 4:**
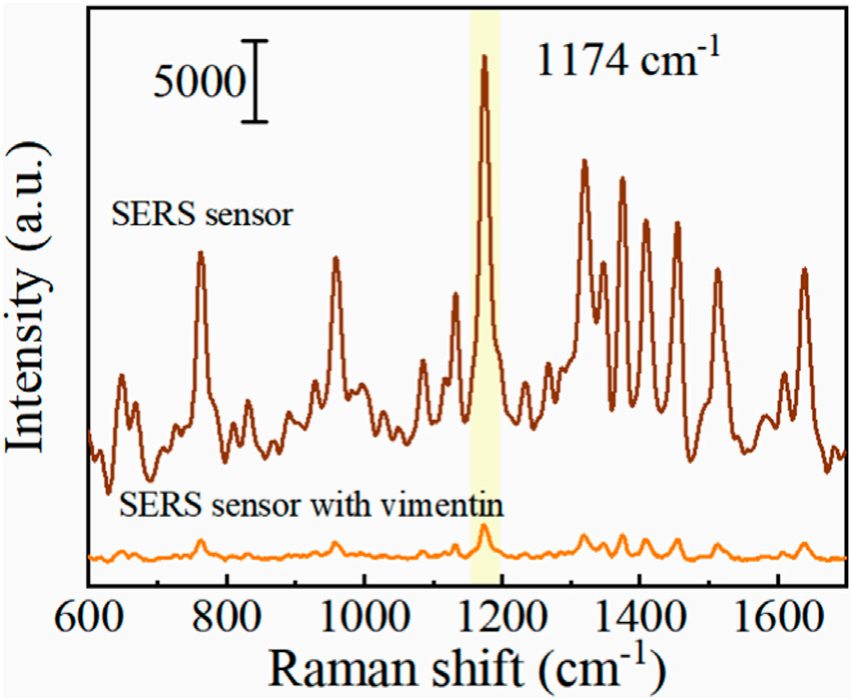
SERS spectra with and without the addition of Vimentin detection.

In the absence of Vimentin, the Vimentin aptamers on the surface of Au/Si CCA arrays binds to the 5-FAM labelled complementary strand on the surface of AuNCs. The Au/Si CCA arrays and AuNCs together provide a SERS “hotspot” that enables the sensor to produce a large SERS enhancement effect. Moreover, the SERS reporter 5-FAM is located within and very close to the “hotspot,” thus generating a 5-FAM SERS spectrum with very high signal intensity. Upon addition of Vimentin, the connection between the Au/Si CCA arrays and AuNCs is broken due to the fact that the Vimentin aptamer on the surface of the Au/Si CCA arrays reacts preferentially with Vimentin and partially de-hybridizes with their complementary strand. As a result, the “hot spot” part of the sensor disappears and the signal intensity of 5-FAM is reduced. The experimental results in Figure 4 are in perfect agreement with the principle. The SERS signal intensity after the addition of Vimentin is significantly lower than that without Vimentin, which indicates the feasibility of this method.

### 3.4 Sensitivity and specificity of the SERS assay

**FIGURE 5:**
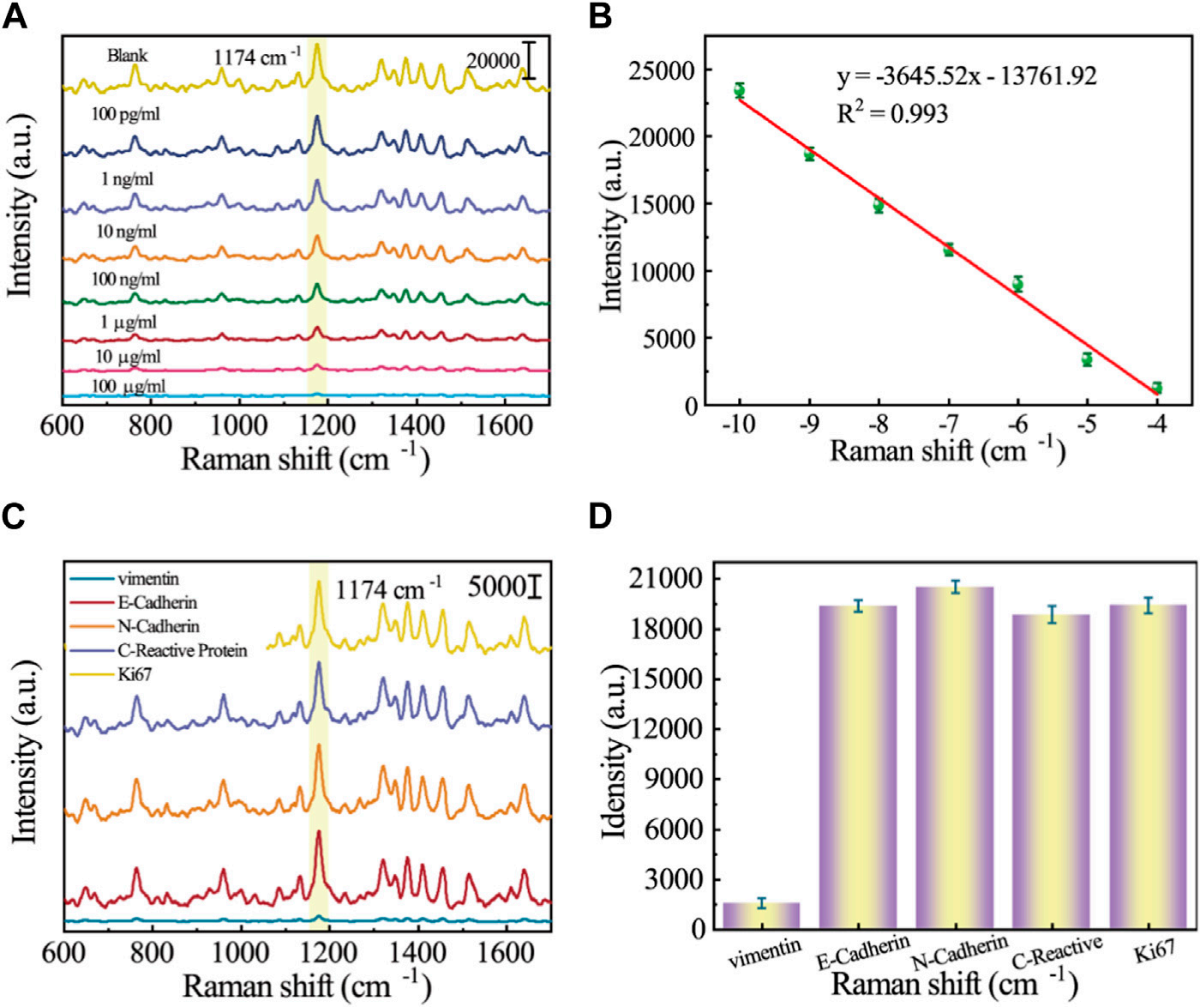
**(A)** SERS spectrograms of protein extracts of different Vimentin concentrations (100 pg/mL–100 mg/mL; **(B)** Corresponding calibration curve for SERS intensity at 1,174 cm^−1^; **(C)** SERS spectrograms for specificity analysis of different proteins (E-Cadherin, N-Cadherin, C-Reactive Protein, Ki67, and Vimentin); **(D)** Corresponding histogram of SERS intensity at 1,174 cm^−1^.

The sensitivity of the SERS sensor to low concentrations of analytes is an important factor in evaluating its performance. Protein extracts of different Vimentin concentrations (100 pg/mL–100 mg/mL) were taken for SERS detection. As can be obtained from Figure 5A, the SERS signal intensity at 1,174 cm^−1^ showed a clear dependence on Vimentin concentration, and remained slightly different from the blank group at Vimentin concentrations as low as 100 pg/mL. Figure 5B shows the scatter plot of its SERS signal intensity versus Vimentin concentration with a linear regression equation of y = −3,645.52x–13,761.92 and *R* ^2^ = 0.993. The LOD of the SERS sensor for Vimentin is as low as 4.92 pg/mL. As shown in Table 2, this method has better detection performance compared to other methods, and was competitive in terms of detection time and LOD. Due to the presence of other proteins in protein extracts from GES-1 cells, in order to assess the specificity of the SERS sensor for Vimentin detection, we introduced different proteins in our experiments (E-Cadherin, N-Cadherin, C-Reactive Protein and Ki67). The results are shown in Figure 5C, where other interfering proteins were unable to affect the SERS spectra of 5-FAM, while Vimentin made the intensity of the spectra significantly weakened, suggesting that the SERS sensor reacts only with Vimentin. Figure 5D shows the corresponding SERS intensity at the characteristic peak of 1,174 cm^−1^. The SERS intensity of Vimentin is much lower than that of other interfering proteins, and it is extremely resistant to interference.

**TABLE 2:** Comparison for determination of different proteins using different methods.

Analyte	Method	Time (min)	LOD	Ref
Cancer antigen 125 (CA-125)	SERS-based lateral flow immunoassay	20	7.182 pg/mL	Xia et al. (2020)
Transferrin	Molecularly imprinted plasmonic nanosensor	15	10^−8^ mol/L	Lv et al. (2016)
Soluble epithermal growth factor receptor (sEGFR)	SERS	240	69.86 pg/mL	Li et al. (2018)
Vimentin	Bead-based immunoassay	30	0.1852 μg/mL	Fhied et al. (2014)
Vimentin	SERS	10	4.92 pg/mL	This work

### 3.5 EMT process confirmed by biological assay

**FIGURE 6:**
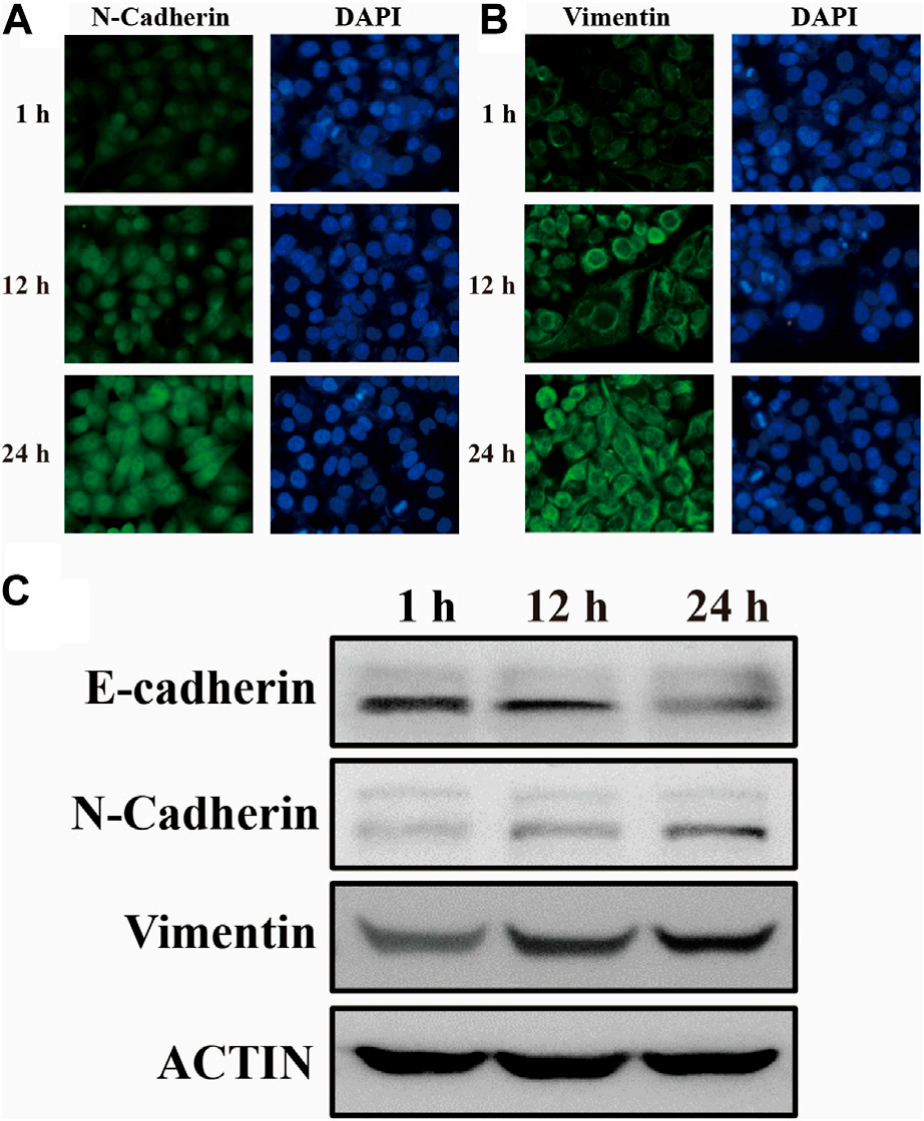
The expression of **(A)** N-calmodulin and **(B)** Vimentin in GES-1 cells after 1 h, 12 h, and 24 h of differentiation was examined by immunofluorescence labelling. **(C)** After 1 h, 12 h, and 24 h of GES-1 cell differentiation, immunoblotting revealed the expression of E-cadherin, N-cadherin, Vimentin, and ACTIN, which was used as an internal control.

It is widely known that epithelial cells undergo a phenotypic change during EMT by losing their cell polarity and epithelial markers like E-calmodulin and acquiring mesenchymal markers like N-calmodulin and Vimentin to create mesenchymal cells (Theys et al., 2011). Among them, the upregulation of vimentin expression was the theoretical basis for this experiment to realize the early discovery of the EMT process. In order to observe the phenotypic and functional changes of human gastric mucosal epithelial cells during EMT more intuitively, GES-1 cells were co-cultured with VCR (1 μg/mL) for 1 h, 12 h, and 24 h (Xue et al., 2012). Subsequently, cellular proteins were extracted and biological experiments were performed. As we expected, the mesenchymal markers N-calmodulin and Vimentin expression levels were progressively upregulated after the antitumor drug VCR induction, reaching the highest level after 24 h (Figures 6A, B). As a further confirmation, the expression levels of E-calmodulin, N-calmodulin, and Vimentin during EMT were validated by Western blotting, and the experimental results were consistent with the immunofluorescence results (Figure 6C). Hence, the results of biological experiments could effectively underpin the SERS spectral analysis of the actual samples in the subsequent EMT process.

### 3.6 Real sample analysis

**FIGURE 7:**
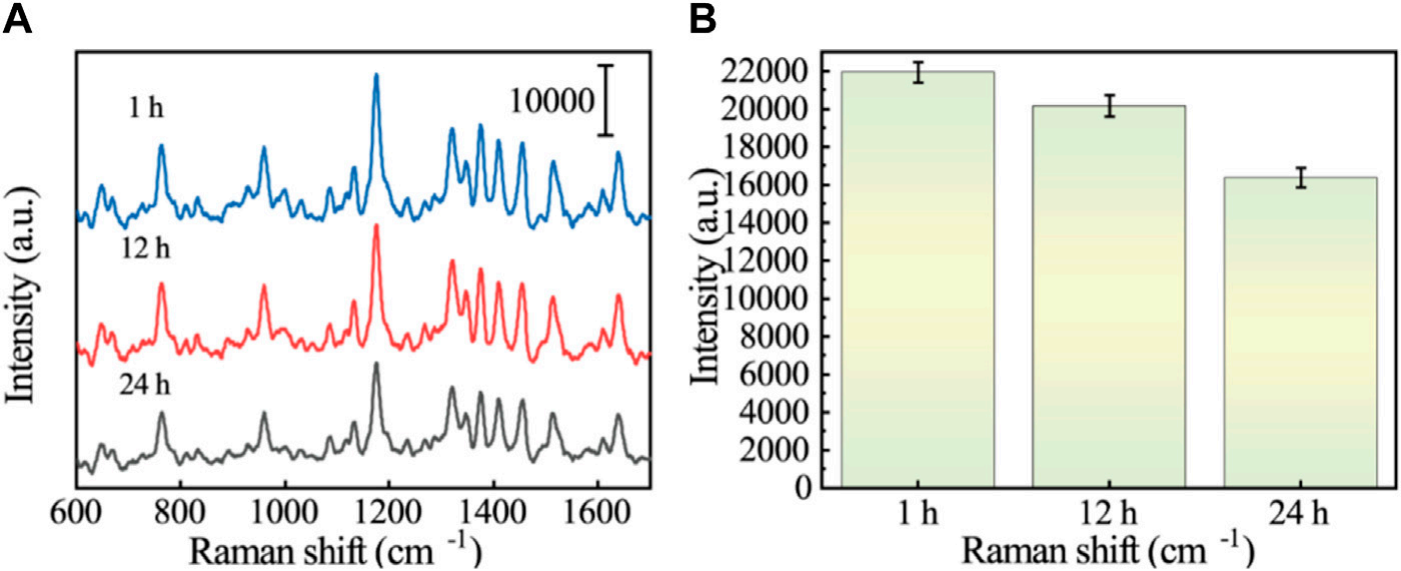
**(A)** SERS spectra of Vimentin in GES-1 cells after treatment with VCR for different times (1 h, 12 h, and 24 h). **(B)** Histogram corresponding to the SERS signal intensity at the 1,174 cm^−1^ characteristic peak.

GES-1 cells were co-cultured with VCR (1 μg/mL) for 1 h, 12 h, and 24 h, and protein was extracted. SERS sensors were used to detect changes in Vimentin during EMT. Figures 7A, B show the SERS spectra of GES-1 cells at different induction times. According to the images, we can see that at the characteristic peak at 1,174 cm^−1^, the SERS intensity gradually decreases with the increase of induction time. The reason is that the Vimentin aptamer on the surface of Au/Si CCA arrays preferentially binds to the Vimentin reaction and partially dehybridizes with its complementary strand, resulting in the connection breakage between Au/Si CCA arrays and AuNCs. As a result, the “hot spot” part of the sensor disappears and the signal intensity of 5-FAM is reduced. Therefore, it can be analyzed that the content of GES-1 cells in VCR co-culture is increased by the content of cellular Vimentin. This demonstrates the reliability of the method.

## 4 Conclusion

In this study, a SERS sensor based on functionalized Au/Si CCA was prepared using the identity-release strategy for the ultra-sensitive detection of Vimentin during GC EMT. The periodic structure of the capture substrate (Au/Si CCA) in this study exhibited good SERS signal enhancement performance and homogeneity. The SERS sensor has a very low detection limit (4.92 pg/mL). The simultaneous identity-release strategy enables the proposed method to have excellent anti-interference ability. GES-1 cells were treated with VCR to simulate the EMT process of GC. The SERS detection results could fully reflect the change of Vimentin in the EMT process. Therefore, this SERS sensor provides a selective, reproducible, and ultra-sensitive method for Vimentin detection, which has great potential for future clinical applications for early GC screening.

## Data Availability

The original contributions presented in the study are included in the article/Supplementary material, further inquiries can be directed to the corresponding author.
